# Retraction Note: An overview on mRNA-based vaccines to prevent monkeypox infection

**DOI:** 10.1186/s12951-024-02693-0

**Published:** 2024-07-09

**Authors:** Mohammad Natami, Amirsasan Gorgzadeh, Arsalan Gholipour, Seyedeh Narges Fatemi, Nima Firouzeh, Maryam Zokaei, Saad Hasan Mohammed Ali, Hadis Kheradjoo, Somayeh Sedighi, Omid Gholizadeh, Shaylan Kalavi

**Affiliations:** 1 Department of Urology, Shahid Mohammadi Hospital, Hormozgan University of Medical Sciences, Bandar Abbas, Iran; 2 Faculty of Pharmacy, Jondi Shapour University, Ahvaz, Iran; 3 Free Researchers, Biotechnology and Nanobiotechnology, Babolsar, Iran; 4 Faculty of Veterinary, University of Tehran, Tehran, Iran; 5 Vector-borne Diseases Research Center, North Khorasan University of Medical Sciences, Bojnurd, Iran; 6 Department of Food Science and Technology, Faculty of Nutrition Science, Food Science and Technology/National Nutrition and Food Technology Research Institute, Shahid Beheshti University of Medical Sciences, Tehran, Iran; 7 College of Dentistry, Al-Mustaqbal University, Babylon, 51001 Iraq; 8 Laboratory Department, Buraimi Hospital, Buraimi, Oman; 9 Medical Science of Mashhad, Mashhad, Iran; 10 AZAD Researchers, Viro & biotche, Tehran, Iran; 11 Department of Clinical Pharmacy, Faculty of Pharmacy, Islamic Azad University of Medical Sciences, Tehran, Iran

**Retraction Note: Journal of Nanobiotechnology (2024) 22:86**

10.1186/s12951-024-02355-1

The Editor-in-Chief has retracted this article because it shows evidence of authorship manipulation. The Editor-in-Chief therefore no longer has confidence in the reliability of the results and conclusions of this article.

The authors disagree with this retraction.

